# Effect of Contextual Interference in the Practicing of a Computer Task in Individuals Poststroke

**DOI:** 10.1155/2020/2937285

**Published:** 2020-07-22

**Authors:** Alice Haniuda Moliterno, Fernanda Vieira Bezerra, Louanne Angélica Pires, Sarah Santos Roncolato, Talita Dias da Silva, Thais Massetti, Deborah Cristina Gonçalves Luiz Fernani, Fernando Henrique Magalhães, Carlos Bandeira de Mello Monteiro, Maria Tereza Artero Prado Dantas

**Affiliations:** ^1^University of West Paulista-UNOESTE-Presidente Prudente, SP, Brazil; ^2^São Paulo State University (Unesp) “Júlio de Mesquita Filho”-Presidente Prudente, SP, Brazil; ^3^Grupo de Pesquisa em Aplicações Tecnológicas em Reabilitação School of Arts, Sciences and Humanities-EACH-University of São Paulo, São Paulo, SP, Brazil; ^4^Laboratory Design and Scientific Writing. Department of Basic Sciences, ABC Faculty of Medicine-Santo André, SP, Brazil

## Abstract

**Objectives:**

Sensory and motor alterations resulting from stroke often impair the performance and learning of motor skills. The present study is aimed at investigating whether and how poststroke individuals and age- and sex-matched healthy controls benefit from a contextual interference effect on the practice of a maze task (i.e., constant *vs.* random practice) performed on the computer.

**Methods:**

Participants included 21 poststroke individuals and 21 healthy controls, matched by sex and age (30 to 80 years). Both groups were divided according to the type of the practice (constant or random) presented in the acquisition phase of the learning protocol. For comparison between the groups, types of practice, and blocks of attempts, the analysis of variance with Tukey's post hoc test (*p* < 0.05) was used.

**Results:**

Poststroke individuals presented longer movement times as compared with the control group. In addition, only poststroke individuals who performed the task with random practice showed improved performance at the transfer phase. Moreover, randomized practice enabled poststroke individuals to perform the transfer task similarly to individuals without any neurological impairment.

**Conclusion:**

The present findings indicated a significant effect of contextual interference of practice in poststroke individuals, suggesting that applying randomized training must be considered when designing rehabilitation protocols for this population.

## 1. Introduction

Stroke is a consequence of blood flow obstruction or hemorrhage in the encephalon [1], which often leads to neurological damage associated with functional limitations and/or disabilities [2] such as cognitive, sensorimotor, and/or language disorders [3].

A variety of rehabilitation programs take advantage of knowledge from motor learning research to optimize the improvement of functional abilities in poststroke individuals [4–6]. Regardless of the approach or intervention technique, the rehabilitation program shall be designed to comprise a set of internal processes associated with practice, training, or experience that results in relatively permanent changes in the performance of motor skills [7, 8].

In a recent systematic review, Shishov et al. [5] pointed out that the very broad types of protocols and measurements used to assess motor learning in poststroke individuals make it difficult to synthesize research findings across studies. Therefore, the authors emphasize the need to promote more studies in order to improve the knowledge of the organization of practice during poststroke rehabilitation. The organization of practice is an important factor to be controlled in motor learning studies, as the type of practice (e.g., constant: always the same task sequence; or random: similar, but with variations on the task sequence) can significantly influence the extent at which motor learning techniques improve performance [9–11]. According to Porter and Magill [12], random tasks reduce performance during training, but enhance retention and transfer as compared with constant schedules, a phenomenon known as the contextual interference (CI) effect. Practicing in high contextual interference conditions (random practice) enforces the individual to use multiple processing strategies to optimize performance during acquisition, whereas no such multiple processing seems necessary for the low contextual interference condition (constant practice). As a consequence, high contextual interference context is associated with improved performance during the retention and transfer phases of the learning process of a motor task (see [13, 14]).

Different theoretical supports have been associated with the CI effect [15]. The first addresses the “forgetting-reconstruction” hypothesis, which consists of short-term forgetting between successive presentations of the same task during random training. This requires the learner to “reconstruct the action plan at each presentation,” which might lead to stronger memory representations [16, 17]. A second theory suggests that the computational models play a crucial role of working memory in the CI effect. It has notably been proposed that motor adaptation occurs via the simultaneous update of a fast process that contributes to fast initial learning but quickly forgetting and a slow process that contributes to long-term retention but slow learning [18, 19]. A third possibility was proposed by Lee and Schweighofer [20], who considered multiple task adaptation. In this model, a single fast process is arranged in parallel with multiple independent slow processes switched via contextual cues. During adaptation, motor errors simultaneously update fast and slow processes in a competitive manner. In a situation in which tasks are interchanged during training, this model predicts that the decay in the fast process due to both time and interference from other tasks leads to the greater update of the slow process. Thus, random schedule training should induce less long-term forgetting than constant schedule training, as proposed in the CI effect.

Some studies have been carried out to address how CI affects motor learning in poststroke individuals [15, 21–23], with findings suggesting a slower change in performance during practice in random schedules as compared with constant schedules (but with retention due to CI effects). On the other hand, in a study with functional magnetic resonance imaging, Boyd et al. [24] showed that practice of a repeated sequence increased cortical activation (suggesting a functional reorganization of the contralesional motor cortex poststroke), whereas random sequence performance did not enhance motor learning.

Although most of the above studies presented a CI effect in agreement with Shishov et al. [5], there is a gap in the motor learning literature in poststroke individuals, as most studies did not use a retention or transfer phase to assess changes in motor performance. Motor learning is basically composed of three phases: (1) acquisition, the phase in which the individual, in a repeated manner, practices how to accomplish a task; (2) retention, defined as the act of retaining previously learned information; and (3) transfer, the moment at which the individual can demonstrate whether or not there was improvement in ability, after modification of the task [11, 25, 26]. Kantak et al. [27] suggested that retention and transfer tests, rather than solely the performance parameter during acquisition, should be implemented to control for transient performance changes and hence to assess motor learning precisely. Moreover, according to Shishov et al. [5], retention tests assess the extent to which performance has improved due to the motor plan that was established during practice (thereby indicating long-lasting changes and the strength of the motor memory built during the acquisition phase), whereas the transfer test would examine the adaptability of the newly acquired motor program. These features are putatively related to the superior performance expected at the retention and transfer phases of random practice schedules as compared to constant practice (i.e. CI effect).

As rehabilitation programs must be focused on gradually improve the performance of a recently practiced motor activity ([27] (which must not only involve improvements during practice but also lead to enhanced skills on retention and transfer), the present study is aimed at contributing to the empirical body of knowledge by addressing whether poststroke individuals benefit from a CI effect, considering all the three stages of the learning process, namely, acquisition, retention, and transfer phases.

For this purpose, we used a motor learning protocol with acquisition, retention, and transfer phases during a simple computer task (maze task) performed by poststroke individuals and by age- and sex-matched healthy controls in order to identify improvements in motor abilities (see [11]). The motor task was chosen to assess distal function motor ability (finger movement), which is considered an important target for poststroke individuals. According to Antunes et al. [28], a computer task that improves the use of the computer keyboard comprises a technological ability that increases poststroke individuals' function and interaction with society (i.e., participation).

Both groups (poststroke and control groups) were divided into two subgroups based on the type of practice: in the subgroup of constant practice, participants performed the acquisition phase by repeating the same maze several times, and in the subgroup using random practice (more CI), the participants performed the acquisition phase using different mazes. As mentioned previously, we were particularly interested in unraveling which type of practice would show better movement time, not only in the acquisition phase but also in considering retention and transfer testing.

We hypothesized that poststroke individuals would perform worse in comparison with individuals from the control group in all phases of the study protocol. Moreover, we hypothesized that individuals who performed the constant practice would show better performance in the acquisition phase of the task as compared to those who practiced randomly, whereas the random practice group would perform better at retention and transfer.

## 2. Methods

### 2.1. Participants

This is a cross-sectional study, approved by the Research Ethics Committee under CAAE number 63123116.7.0000.5515. All participants signed the informed consent form. As the inclusion criterion of this study, individuals were required to present hemiparesis as a sequel of stroke for a minimum of 2 months. The exclusion criteria consisted of those who did not understand the proposed task after three attempts and those with comorbidities that precluded the performance of the activity (skeletal muscle deformity) [29].

Data collection was performed at a physiotherapy clinic in Presidente Prudente/SP (Brazil), and 42 individuals (between 30 and 80 years of age) were evaluated: 21 individuals after stroke (stroke group; SG) and 21 health individuals (control group; CG), matched by sex and age.

### 2.2. Instruments and Procedures

For characterization of the sample, the following instruments were applied: the Orpington Scale to classify the severity and estimate the prognosis of functional improvement [30, 31], the Fugl Meyer Evaluation Scale (FMS) to assess motor and sensory impairment [32], and the Berg Balance Scale to evaluate balance ability [33]. In addition, hand muscle function was evaluated through palmar grip dynamometry (Saehan® brand dynamometer) [34], and unilateral manual dexterity was measured with the Box and Block Test (BBT) [35].

#### 2.2.1. Maze Task

**Figure 1:**
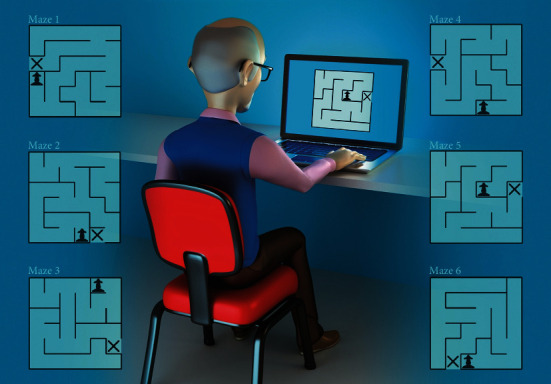
Representative example of a participant performing the maze task and the six different mazes used in the study.

The main tool used in the present study to evaluate motor learning was the maze task, which consists of six different mazes. The task was performed on a notebook positioned on a table, and the participants sat in a chair so that they would feel comfortable to perform the task on the computer (Figure 1). Participants were then given the instruction to perform the task with their preferred upper limb, which was also the limb commonly used to perform fine motor activities in daily life (all individuals chose the nonaffected arm). In addition, the participants also received information about the activity that they should follow the maze path as fast as possible until the end of the maze (signaled with an “X”), using the arrows displayed on the computer (up, down, right, and left), requiring perception and spatial memory during the execution [11]. When the participant finished each trial, a feedback message with the information about the time (in seconds) used to complete the maze was displayed at the monitor screen, thereby providing participants with the knowledge of their performance.

**Figure 2:**
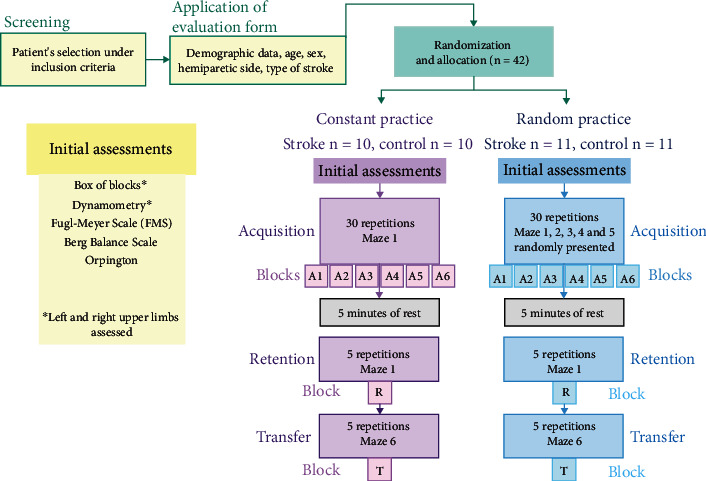
Depiction of the experimental protocol, showing sample selection and the presentation of the 6 different mazes according to the type of practice (constant *vs.* random) during acquisition, retention, and transfer phases of the training.

In the present protocol, the individuals in the SG and CG were divided into subgroups according to the type of task (random or constant practice) (Figure 2). In the acquisition phase, individuals who performed constant practice carried out 30 repetitions of Maze 1, whereas the participants of the random practice subgroup randomly performed 30 repetitions involving 5 different mazes (Mazes 1, 2, 3, 4, and 5, which were randomly presented). After 5 min of rest, retention was performed in which all individuals performed Maze 1 five times (the same maze used in constant practice acquisition and also part of the random practice). This protocol was used by Prado et al., [11], and Maze 1 was chosen for the retention test for both groups in order to allow appropriate comparisons between types of practice (constant *vs.* random). For the retention phase, 5 repetitions of Maze 1 were performed in constant and random practices. In the transfer phase, participants performed 5 attempts of maze 6 (new maze, i.e., not presented before), with no resting time between retention and transfer. In summary, all participants performed 30 attempts of acquisition, 5 attempts for retention, and 5 attempts for transfer. These attempts were divided into blocks with five trials each, yielding 6 blocks of acquisition (A1 to A6), 1 block of retention (R) and 1 block of transfer (T) (see Figure 2).

In this study, three important choices should be explained:

1. Upper limb and fine movements: in this study, all participants used the more functional upper limb (i.e., participants of the control group used the preferred arm whereas poststroke participants used the nonaffected arm). For the poststroke participants, the nonaffected arm corresponded to the dominant side after stroke (commonly used for daily manual activities). In a recent systematic review, Shishov et al. [5] summarized a set of studies in which learning associated with a given intervention was inferred by examination of the nonaffected limb, which seems to be the appropriate choice to distinguish motor execution impairments due to hemiparesis (as the pathological condition, *per se,* might mask motor learning) (see [36]).
2. Task used: to verify motor learning, we used a computer maze task which has been already used for similar studies on cerebral palsy [11] and Duchenne muscular dystrophy [37, 38]. According to Souza et al. [39], this is a simple task with the possibility of evaluating various neuropsychological issues, such as executive function, implicit memory, and spatial learning. The maze activity continuously challenges the individual's ability to process the new information related to the execution of the movement and the motor planning, since this task involves mechanisms such as memory, perception, sensory processing, feedback circuits, and effective execution of the movement [40–44]. Moreover, examining a simple task in motor learning is recommended to avoid movement compensations and was used in most of the previous studies [5].
3. Computer task: another important choice for this protocol was the use of a computer task. Assessing the ability of poststroke patients to use a keyboard is important as effective use of computer tools is essential for poststroke patients in order to socialize, access their bank account, have fun, and so on. When considering new approaches for rehabilitation, technology has become an innovative resource, as it establishes a stimulating and pleasurable relationship between individuals, assists with functional evaluation, and possibly improves day-to-day function [45–47]. Thus, the use of a technological device (i.e., computer keyboard) provides new forms of evaluation and intensifies the rehabilitation process, with the aim of minimizing dysfunction in participation [48]. According to Wearden [49] and Milot et al. [50]; the individual tends to pay more attention when challenged by a task with a higher index of difficulty

### 2.3. Data Analysis

The subgroups of poststroke individuals (constant *vs* random practice) were compared with Student *t*-test to investigate for possible differences in age, FMS, Berg scale, dynamometry, BBT, and date of injury, whereas a chi-square test was used to compare sex, hemiparetic side, and type of stroke. A Bonferroni post hoc test was used to compare the proportions in the intragroup and intergroup analyses of type of stroke.

The results of motor learning analyses were obtained through blocks of five trials for all phases of the study (acquisition, retention, and transfer). We used an Analysis of Variance (ANOVA) with the factors “group” (Stroke *vs.* Control), “type of practice” (Constant *vs.* Random), and “block,” with repeated measures on the factor (“block”). In addition, for the factor block, separate comparisons were made for the study phases: the first block of acquisition versus the last block of acquisition (A1 versus A6), the last block of acquisition versus the retention block (A6 versus R), and the last block of acquisition versus the transfer block (A6 versus T). To identify the differences, the least significant difference (LSD) post hoc test was performed. Partial eta-squared (*η*_*p*_^2^) was reported to measure the effect size and was interpreted as small (effect size > 0.01), medium (effect size > 0.06), or large (effect size > 0.14) [51]. A linear regression analysis was carried out considering the improvement in time in seconds from the first to the final block of the acquisition to identify which factors (age, sex, hemiparetic side, date of injury in months, type of stroke, FMS, right-side BBT, left-side BBT, right-side dynamometry, left-side dynamometry, Berg Scale, and Orpington Scale) influenced the improvement in performance. A significance level of *p* < 0.05 was considered.

## 3. Results

Table 1 depicts the characterization of the participants of this study, indicating the homogeneity of the sample as assessed by the absence of significant differences between the factors (age, sex, hemiparetic side, and type of stroke). Individual data from each patient (such as age, gender, hemiparesis, months from lesion, type of lesion, Fugl Meyer Scale, box of blocks, dynamometry, and Berg and Orpington Scales) are attached as Supplementary Material (available here).

For the analysis of performance, separate ANOVAs were performed for each phase of the study, presented below.

### 3.1. Acquisition

#### 3.1.1. Main Effects

A main effect for the group was found (*F*(1, 35) = 19.3, *p* < 0.001, *η*_*p*_^2^ = 0.36), in which the SG presented worse performance (*M* = 39 s) when compared with the CG (*M* = 15 s).

#### 3.1.2. Interactions

**Figure 3:**
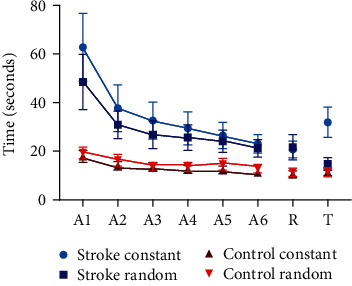
Graphical representation of the mean and standard error of the performance of groups in all phases of the study, in both types of practice. A1 = acquisition 1; A2 = acquisition 2; A3 = acquisition 3; A4 = acquisition 4; A5 = acquisition 5; A6 = acquisition 6; R = retention; T = transfer.

The results are presented in Figure 3. Considering the acquisition, there was a significant interaction for blocks by groups (*F*(1, 35) = 16.7, *p* < 0.001, *η*_*p*_^2^ = 0.32). This result demonstrates that the groups improved performance from the first (*M* = 37 s) to the final block (*M* = 17 s) of the acquisition; however, the post hoc test demonstrated that this performance improvement was only for the SG (*M* = 56 s for 22 s, *p* < 0.001), since the CG did not present significant improvement (*M* = 19 s for 12 s, *p* = 0.155). No effects or interactions for type of practice were found.

### 3.2. Retention

#### 3.2.1. Main Effects

The main effect for groups remained present (*F*(1, 35) = 12.6, *p* = 0.001, *η*_*p*_^2^ = 0.27), in which the SG presented a longer time (*M* = 22 s) than the CG (*M* = 12 s).

#### 3.2.2. Interactions

There were no effects or interactions for blocks, suggesting that the performance acquired in the practice of the task was retained. Similarly, no effects or interactions for type of practice were found.

### 3.3. Transfer

#### 3.3.1. Main Effects

A main effect for groups remained (*F*(1, 35) = 14.2, *p* = 0.001, *η*_*p*_^2^ = 0.29). This result demonstrates that the SG presented a longer time to perform the task (*M* = 23 s) when compared with the CG (*M* = 12 s).

#### 3.3.2. Interactions

No effect was found for blocks; however, there were interactions for blocks by type of practice (*F*(1, 35) = 22.4, *p* < 0.001, *η*_*p*_^2^ = 0.39) and blocks by type of practice by groups (*F*(1, 35) = 14.2, *p* = 0.001, *η*_*p*_^2^ = 0.29). The post hoc test showed that the constant SG presented a worse performance in the transfer test (*M* = 32 s) when compared with the final acquisition block (*M* = 23 s), while the random SG improved performance in the transfer test (*M* = 14 s), in relation to the final acquisition block (*M* = 21 s). In the CG, all participants maintained the same performance, regardless of the type of practice. That is, only the SG with constant practice did not present transfer of performance.

Also, a (marginal) interaction between groups and type of practice (*F*(1, 35) = 3.13, *p* = 0.085, *η*_*p*_^2^ = 0.08). The post hoc test presented an interesting result in the transfer phase: the random SG did not demonstrate differences in relation to the CG (*M* = 18 s in the SG; *M* = 12 s in the CG), and there were differences between only the groups that performed the constant practice (*M* = 28 s in the SG; M =11 s in the CG).

### 3.4. Regression Analysis

To understand which factors may influence performance improvement during practice, a regression analysis was performed between the improvement in movement time from the first to the last acquisition block (*Δ* between blocks). The analysis revealed a significant regression model for the CG (*F*(1, 20) = 5.3, *p* = 0.032, *r*^2^ = 0.22), resulting in the following equation: improved movement time = –0.202 × points in the right-side BBT. In other words, the points in the BBT on the right side influenced the improvement during the practice of the task. In the SG, there was no significant regression model.

## 4. Discussion

The present study assessed motor learning in poststroke individuals as compared with healthy controls using a computer maze as the movement task. Individuals in the SG took more time to perform the maze task as compared with the CG in most stages of the practice. This finding is interpreted as a consequence of the lesion in brain areas (e.g., the cerebral cortex) [52] that might negatively affect the planning and execution of motor tasks that requires spatial memory and organization [39]. It is well known that the ability to find the right way into a novel or familiar environment (such as in the maze task) is a multifactorial function [53]. General deficiencies in attention, memory, and perceptual skills lead to an inability to find the correct path in known and unknown places, in addition to preclude the execution of the task in a short time [54].

Previous research evaluated motor learning using a computer maze task in individuals with Duchenne muscular dystrophy [37], while Prado et al. [11] and De Paula et al. [55] used the same task to evaluate individuals with cerebral palsy. Similarly, Possebom et al. [56] and Menezes et al. [57] studied individuals with Down syndrome, Santos et al. [58] investigated the motor learning effects in individuals institutionalized in shelters, and Souza et al. [39] involved university students in a very similar maze protocol. All the mentioned studies verified that participants in the experimental group (i.e., with the pathology/condition under study) presented a longer time of task execution as compared to their paired controls. The authors interpreted that the longer time was probably associated with loss of function and incapacity inherent to the pathologic conditions, which can also be found in the nonaffected arm of poststroke individuals [59].

The present study also showed that poststroke individuals demonstrated significant improvement in performance during acquisition, reducing the movement time from the first to the final block of the acquisition phase, regardless of the type of practice. Additionally, in the present study, the SG demonstrated retention of practice after the training period, regardless of the type of practice (random or constant). These findings are somewhat different from previous reports that evidenced a better performance during acquisition with constant practice as compared to ramdom practice [7, 14, 60, 61] and also differ from previous studies that showed the application of random practice as an essential feature to improve performance at the retention phase [62]. Such an apparent contradiction between the present and some previous results is probably associated with the specificity of the task used in the present study, as training a very simple task as the computer maze might had been sufficient for this population to improve performance during the acquisition and retention, regardless of the type of practice [62–64].

On the other hand, the general improvement in performance of the SG group during acquisition is in line with the study of Malheiros [38], which reported participants with Duchenne muscular dystrophy performed better than typically developed individuals at the final block of the computer maze task as compared to the first block of the acquisition phase. Additionally, the present study and that from Malheiros [38] observed that the control group did not improve performance during the motor learning protocol (acquisition, retention, and transfer phases), which is probably associated with the nature of the task, which is very easy to perform and probably not challenging for participants without cognitive and/or sensorimotor impairments.

In the present study, poststroke participants that trained under random practice showed effective motor learning at the transfer phase, whereas those that trained under constant practice did not. Such a better performance at the transfer phase for random practice as compared to constant practice might be associated with the fact that the randomly performed training required motor reorganization with multiple processing strategies [14, 15, 65] in the recruitment of upper limb muscles at each attempt of the acquisition phase. Consequently, the participants subjected to random practice could probably adapt to these changes [21] and hence demonstrated a superior performance at the transfer phase. Furthermore, it is necessary to constantly create a plan of action in the face of the frequent changes in the task [17], which favors the performance of the random practice group at the transfer phase, whereas the participants with constant practice were not adapted to modifications and hence could not reorganize the plan of action necessary for transfer. This result has a strong practical implication, as the main goal of practicing motor tasks (acquisition phase), especially for individuals in the rehabilitation process, is the transfer of the improved performance to similar tasks, which is indeed the most difficult goal to reach and rarely addressed in motor learning research [66].

Lastly, and rather interesting, no difference could be observed between the performance of the SG (trained with random practice) and the CG (trained with both random and constant practice) at the transfer phase. This finding indicates that the randomized practice enabled poststroke individuals to perform the transfer task similarly to individuals without any neurological impairment. This finding is probably due to CI effects, so the reorganization of motor action necessary at each attempt demanded the participants to constantly apply task-appropriate motor strategies, which is known to facilitate learning [67]. In the case of poststroke individuals, this may possibly improve motor ability to perform as well as healthy subjects [17, 21]. Further studies must be carried out (e.g., using different tasks) to investigate CI effects in poststroke individuals, to confirm whether considering random practice as an important component of intervention programs shall be indicated for this population.

## 5. Limitations and Future Directions

One of the limitations of the present study was that the participants with stroke performed the maze task with their nonaffected arm (which was eventually the “original” nondominant arm before stroke for some participants), whereas the healthy control participants performed the task with their dominant arm. Although this might have influenced the present results somehow, it is worth noting that most of the participants of the SG were chronic patients (67.7 months after injury for the constant practice group and 38.1 months for the random practice group). Thus, the poststroke participants were used to perform daily tasks using their nonaffected arm, regardless of their original handedness.

Additionally, the present study involved a relatively small number of participants; i.e., a larger sample could have provided more detailed data about time of injury, strength, and comparison between sides (right and left). Regardless of the small number of participants, the large effect sizes associated with the present results suggest the sample was large enough to provide reliable results.

This study used a short-term protocol and a specific computer maze task, so that the present results cannot be generalized to different tasks and/or long-term effects. Moreover, the present study did not directly investigate the neurological or physiological mechanisms associated with the present findings. Although previous studies suggest mechanisms such as cortical reorganization, adaptive changes in the functional organization of the motor system and plasticity due to motor learning intervention (e.g., [68]) might be associated with the behavioral outcomes observed here; the effects of the short-term protocol used in the present study might only be associated with these mechanisms in a speculative way. Thus, considering the promising results, future studies with long-term learning protocols and direct neurological assessments are warranted in order to provide more conclusive results about the mechanisms and the clinical applicability associated with motor learning by random practice in poststroke individuals.

Finally, further clinical assessments (besides gender, hemiplegic side, age, and functional classification), such as visual and cognitive measurements, could have been useful to better characterize the sample and interpret the results. Nevertheless, despite the limitation pointed above, the present results suggest that motor training of poststroke patients can be more effective with the implementation of random practice rather than constant practice. Thus, the findings presented herein provide useful insights on tasks for fine movements of the upper limbs, which are important for the accomplishment of activities of daily living and are generally impaired in poststroke patients [63].

## 6. Conclusion

Poststroke individuals presented lower performance on the maze task as compared with healthy controls. In addition, only poststroke individuals who performed the task with random practice showed improved performance at the transfer phase, which suggests an effect of contextual interference of practice. Moreover, randomized practice enabled poststroke individuals to perform the transfer task similarly to individuals without any neurological impairment. This finding might be considered an indication of the importance of applying randomized training in the rehabilitation of poststroke individuals, as the type of practice in the acquisition phase significantly influenced overall motor learning.

## Figures and Tables

**Table 1:** Identification characteristics and clinical aspects of the individuals participating in the study, with values expressed as the mean and standard deviation (*n* = 39).

	Experimental			Control		
	Stroke constant (*n* = 10)	Stroke random (*n* = 11)	*p*	Control constant (*n* = 10)	Control random (*n* = 11)	*p*
Age (years)	55.9 ± 12.6	52.7 ± 11	0.240	56.2 ± 12.7	52.6 ± 10.6	0.487
Box of blocks (R) (units)	26.8 ± 20.2	29.9 ± 21.2	0.162	73.7 ± 8.2	70.4 ± 10.7	0.398
Box of blocks (L) (units)	24.2 ± 14.4	23.2 ± 22.5	0.270	71.4 ± 8.1	66.4 ± 10.7	0.353
Dynamometry (R) (kg)	29.2 ± 26.1	36.3 ± 23.5	0.762	71.3 ± 15.0	68.3 ± 22.8	0.200
Dynamometry (L) (kg)	26.2 ± 19.9	27.7 ± 29.3	0.043^∗^	67 ± 17	62.3 ± 22.8	0.374
Time of injury (months)	67.7 ± 108.5	38.1 ± 72	0.684	—	—	
FMS (points)	74.2 ± 14.4	69 ± 14	0.377	—	—	
BBS (points)	39.9 ± 11.9	46.1 ± 7.5	0.083	—	—	
Orpington (points)	3.4 ± 1.5	3.3 ± 1.1	0.403	—	—	
Sex (*n*)						
Masculine	6	5	0.500	7	7	0.562
Feminine	3	4		3	4	
Hemiparetic side (*n*)						
Right	3	3	0.690	—	—	
Left	6	6		—	—	
Side used for task	Opposite of paresis			Dominant arm		
Dominant side						
Right	—	—		8	9	
Left	—	—		2	2	
Type of stroke (*n*)						
Ischemic	4	8	0.066	—	—	
Hemorrhagic	5	1		—	—	

*Note.* R = right; L = left; FMS = Fugl Meyer Evaluation Scale; BBS = Berg Balance Scale; *n* = number of individuals. ^∗^Level of significance (*p* < 0.05).

## Data Availability

All relevant data are within the paper. Any other data concerning the results communicated herein will be freely available upon request.

## Supplementary Materials

Supplementary Materials "Demographic data for each patient included in the study".

## References

[1] Naz L., Mushtaq S. (2016). Risk factors of transient ischemic attack: an overview. *International Journal of Biological Research*.

[2] Silva S. M., Corrêa F. I., Faria C. D. C. M., Corrêa J. C. F. (2015). Psychometric properties of the stroke specific quality of life scale for the assessment of participation in stroke survivors using the rasch model: a preliminary study. *Journal of Physical Therapy Science*.

[3] Al Kasab S., Lynn M. J., Turan T. N. (2017). Impact of the new American Heart Association/American Stroke Association definition of stroke on the results of the Stenting and Aggressive Medical Management for Preventing Recurrent Stroke in Intracranial Stenosis Trial. *Journal of Stroke and Cerebrovascular Diseases*.

[4] Langhorne P., Bernhardt J., Kwakkel G. (2011). Stroke rehabilitation. *The Lancet*.

[5] Shishov N., Melzer I., Bar-Haim S. (2017). Parameters and measures in assessment of motor learning in neurorehabilitation; a systematic review of the literature. *Frontiers in Human Neuroscience*.

[6] Silva T. D., Raimundo R. D., Ferreira C. (2013). Comparison between the six-minute walk test and the six-minute step test in post stroke patients. *International Archives of Medicine*.

[7] Schmidt R. A. (1975). A schema theory of discrete motor skill learning. *Psychological Review*.

[8] Schmidt R. A. (1988). *Motor Control and Learning: A Behavioral Emphasis*.

[9] Hornby T. G., Straube D. S., Kinnaird C. R. (2015). Importance of specificity, amount, and intensity of locomotor training to improve ambulatory function in patients poststroke. *Topics in Stroke Rehabilitation*.

[10] Pinheiro J. P., Marques P. G., Tani G., Corrêa U. C. (2015). Diversification of motor skills rely upon an optimal amount of variability of perceptive and motor task demands. *Adaptive Behavior*.

[11] Prado M. T. A., Fernani D. C. G. L., Silva T. D. D., Smorenburg A. R. P., Abreu L. C., Monteiro C. B. M. (2017). Motor learning paradigm and contextual interference in manual computer tasks in individuals with cerebral palsy. *Research in Developmental Disabilities*.

[12] Porter J. M., Magill R. A. (2010). Systematically increasing contextual interference is beneficial for learning sport skills. *Society of Sports Sciences*.

[13] Schmidt R. A., Lee T. D. (1999). *Motor Control and Learning: A Behavioral Emphasis*.

[14] Shea J. B., Morgan R. L. (1979). Contextual interference effects on the acquisition, retention, and transfer of a motor skill. *Journal of Experimental Psychology: Human Learning & Memory*.

[15] Schweighofer N., Lee J. Y., Goh H. T. (2011). Mechanisms of the contextual interference effect in individuals poststroke. *Journal of Neurophysiology*.

[16] Lee T. D., Magill R. A. (1983). The locus of contextual interference in motor-skill acquisition. *Journal of Experimental Psychology Human Perception & Performance*.

[17] Lee T. D., Magill R. A. (1985). Can forgetting facilitate skill acquisition?. *Advances in Psychology*.

[18] Joiner W. M., Smith M. A. (2008). Long-term retention explained by a model of short-term learning in the adaptive control of reaching. *Journal of Neurophysiology*.

[19] Smith M. A., Ghazizadeh A., Shadmehr R. (2006). Interacting adaptive processes with different timescales underlie short-term motor learning. *PLoS Biology*.

[20] Lee J. Y., Schweighofer N. (2009). Dual adaptation supports a parallel architecture of motor memory. *Journal of Neuroscience*.

[21] Hanlon R. E. (1996). Motor learning following unilateral stroke. *Archives of Physical Medicine and Rehabilitation*.

[22] Imam B., Jarus T. (2014). Virtual reality rehabilitation from social cognitive and motor learning theoretical perspectives in stroke population. *Rehabilitation Research and Practice*.

[23] Pohl P. S., McDowd J. M., Filion D., Richards L. G., Stiers W. (2016). Implicit learning of a motor skill after mild and moderate stroke. *Clinical Rehabilitation*.

[24] Boyd L. A., Vidoni E. D., Wessel B. D. (2010). Motor learning after stroke: is skill acquisition a prerequisite for contralesional neuroplastic change?. *Neuroscience Letters*.

[25] Emanuel M., Jarus T., Bart O. (2008). Effect of focus of attention and age on motor acquisition, retention, and transfer: a randomized trial. *Physical Therapy*.

[26] Mulder T. A. (1991). A process-oriented model of human motor behavior: toward a theory-based rehabilitation approach. *Physical Therapy*.

[27] Kantak S. S., Wistein C. J. (2012). Learning–performance distinction and memory processes for motor skills: A focused review and perspective. *Behavioral Brain Research*.

[28] Antunes T. P., Oliveira A. S., Crocetta T. B. (2017). Computer classes and games in virtual reality environment to reduce loneliness among students of an elderly reference center: study protocol for a randomised cross-over design. *Medicine (Baltimore)*.

[29] Martins F. P. A., Massetti T., Crocetta T. B. (2019). Analysis of motor performance in individuals with cerebral palsy using a non-immersive virtual reality task - a pilot study. *Neuropsychiatric Disease and Treatment*.

[30] Celik C., Aksel J., Karaoglan B. (2009). Comparison of the Orpington Prognostic Scale (OPS) and the National Institutes of Health Stroke Scale (NIHSS) for the prediction of the functional status of patients with stroke. *Disability and Rehabilitation*.

[31] Studenski S. A., Wallace D., Duncan P. W., Rymer M., Lai S. M. (2001). Predicting stroke recovery: three- and six-month rates of patient-centered functional outcomes based on the Orpington Prognostic Scale. *Journal of the American Geriatrics Society*.

[32] Fugl-Meyer A. R., Jaasko L., Leyman I., Olsson S., Steglind S. (1975). The post-stroke hemiplegic patient: 1. a method for evaluation of physical performance. *Scandinavian Journal of Rehabilitation Medicine*.

[33] Liljehult M. M., Buus L., Liljehult J., Rasmussen B. K. (2017). Walking ability in patients with glioblastoma: prognostic value of the Berg Balance Scale and the 10-meter walk test. *Journal of Neuro-Oncology*.

[34] Klidjian A. M., Foster K. J., Kammerling R. M., Cooper A., Karran S. J. (1980). Relation of anthropometric and dynamometric variables to serious postoperative complications. *BMJ*.

[35] Mathiowetz V., Volland G., Kashman N., Weber K. (1985). Adult norms for the Box and Block Test of manual dexterity. *American Journal of Occupational Therapy*.

[36] Boyd L. A., Winstein C. J. (2004). Providing explicit information disrupts implicit motor learning after basal ganglia stroke. *Learning & Memory*.

[37] Capelini C. M., da Silva T. D., Tonks J. (2017). Improvements in motor tasks through the use of smartphone technology for individuals with Duchenne muscular dystrophy. *Neuropsychiatric Disease and Treatment*.

[38] Malheiros S. R. P., Silva T. D., Favero F. M. (2015). Computer task performance by subjects with Duchenne muscular dystrophy. *Neuropsychiatric Disease and Treatment*.

[39] Souza D. E., França F. R., Campos T. F. (2006). Teste de labirinto: instrumento de análise na aquisição de uma habilidade motora. *Revista Brasileira de Fisioterapia*.

[40] Choshi K. (2017). Motor learning as an ill-posed problem. *Revista Paulista de Educação Física*.

[41] Dancause N., Ptito A., Levin M. F. (2002). Error correction strategies for motor behavior after unilateral brain damage: short-term motor learning processes. *Neuropsychologia*.

[42] Grafton S. T., Mazziota J. C., Presty S., Fristow K. J., Frackowiac R. S. J., Phelps M. E. (1992). Functional anatomy of human procedural learning determined with regional cerebral blood flow and PET. *Journal of Neuroscience*.

[43] Mattar A. A. G., Gribble P. L. (2005). Motor learning by observing. *Neuron*.

[44] Nixon P. D., McDonald K. R., Gough P. M., Alexander I. H., Passingham R. E. (2004). Cortico-basal ganglia pathways are essential for the recall of well-established visuomotor associations. *European Journal of Neuroscience*.

[45] Merians A. S., Fluet G., Tunik E., Qiu Q., Saleh S., Adamovich S. (2014). Movement rehabilitation in virtual reality from then to now: how are we doing?. *International Journal on Disability and Human Development*.

[46] Pompeu J. E., Alonso T. H., Masson I. B., Pompeu S. M. A. A., Pasin C. T. (2014). Os efeitos da realidade virtual na reabilitação do acidente vascular encefálico: uma revisão sistemática. *Motricidade*.

[47] Wuang Y. P., Chiang C. S., Su C. Y., Wang C. C. (2011). Effectiveness of virtual reality using Wii gaming technology in children with Down syndrome. *Research in Developmental Disabilities*.

[48] Cameirão M. S., Badia S. B., Duarte E., Verschure P. F. M. J. (2010). Neurorehabilitation using the virtual reality-based Rehabilitation Gaming System: methodology, design, psychometrics, usability and validation. *Journal of Neuroengineering and Rehabilitation*.

[49] Wearden J. H. (2004). Decision processes in models of timing. *Acta Neurobiologiae Experimentalis*.

[50] Milot M. H., Marchal-Crespo L., Green C. S., Cramer S. C., Reinkensmeyer D. J. (2010). Comparison of error-amplification and haptic-guidance training techniques for learning of a timing-based motor task by healthy individuals. *Experimental Brain Research*.

[51] Lakens D. (2013). Calculating and reporting effect sizes to facilitate cumulative science: a practical primer for t-tests and ANOVAs. *Frontiers in Psychology*.

[52] Subramaniam S., Wan-Ying C. H., Bhatt T. (2014). Effect of dual tasking on intentional vs. reactive balance control in people with hemiparetic stroke. *Journal of Neurophysiology Published*.

[53] Brunsdon R., Nickels L., Coltheart M. (2007). Topographical disorientation: towards an integrated framework for assessment. *Neuropsychological Rehabilitation*.

[54] Carelli L., Rusconi M. L., Scarabelli C., Stampatori C., Mattioli F., Riva G. (2011). The transfer from survey (map-like) to route representations into virtual reality mazes: effect of age and cerebral lesion. *Journal of Neuroengineering and Rehabilitation*.

[55] de Paula J. N., de Mello Monteiro C. B., da Silva T. D. (2017). Motor performance of individuals with cerebral palsy in a virtual game using a mobile phone. *Disability and Rehabilitation Assistive Technology*.

[56] Possebom W. F., Silva T. D., Ré A. H. N. (2016). Maze computer performance in Down syndrome. *Temas sobre Desenvolvimento*.

[57] Menezes L. D. C., Gomes K. S. C., Massetti T. (2015). Motor learning in mobile (cell phone) device in Down syndrome patients - pilot project. *Medical Express*.

[58] Santos C. M. S., Rodrigues M. M., Fernani D. C. G. L., Freire A. P. C. F., Monteiro C. B. M., Prado M. T. A. (2017). Motor learning in children and adolescents institutionalized in shelters. *Fisioterapia em Movimento*.

[59] Ekstrand E., Lexell J., Brogårdh C. (2016). Grip strength is a representative measure of muscle weakness in the upper extremity after stroke. *Topics in Stroke Rehabilitation*.

[60] Barreiros J. (2006). Interferência e variabilidade na aprendizagem. *Revista Brasileira Educação Física Especial*.

[61] Lee T., Genovese E. D. (1988). Distribution of practice in motor skill acquisition: learning and performance effects reconsidered. *Research Quarterly for Exercise and Sport*.

[62] Wright D., Verwey W., Buchanen J., Chen J., Rhee J., Immink M. (2016). Consolidating behavioral and neurophysiologic findings to explain the influence of contextual interference during motor sequence learning. *Psychonomic Bulletin & Review*.

[63] Park H., Shweighofer N. (2017). Nonlinear mixed-effects model reveals a distinction between learning and performance in intensive reach training post-stroke. *Journal of Neuroengineering and Rehabilitation*.

[64] Sheikh K. M., Kolahdouzi S., Pour H. F., Rad M. T. A. (2017). Effect of combined mental and physical training on the targeting accuracy of patients with multiple sclerosis. *Journal of Research & Health*.

[65] Guadagnoli M. A., Lee T. D. (2004). Challenge point: a framework for conceptualizing the effects of various practice conditions in motor learning. *Journal of Motor Behavior*.

[66] Walter C. S., Hengge C. R., Lindauer B. E., Schaefer S. Y. (2019). Declines in motor transfer following upper extremity task-specific training in older adults. *Experimental Gerontology*.

[67] Kearney P. E., Judge P. (2017). Successful transfer of a motor learning strategy to a novel sport. *Perceptual and Motor Skills*.

[68] Winstein C., Lewthwaite R., Blanton S. R., Wolf L. B., Wishart L. (2014). Infusing motor learning research into neurorehabilitation practice: a historical perspective with case exemplar from the accelerated skill acquisition program. *Journal of Neurologic Physical Therapy: JNPT*.

