# Supplementary material for: Effect of Contextual Interference in the Practicing of a Computer Task in Individuals Poststroke
Source: Biomed Res Int. 2020 Jul 22;2020:2937285. doi: 10.1155/2020/2937285 (PMC7396124; doi:10.1155/2020/2937285)
Supplement: Supplementary Materials — "Demographic data for each patient included in the study". [file 2937285.f1.docx]

Table S1: Data of assessments of each participant in the Poststroke group.

| Subject | Type of practice | Age | Gender | Hemiparesis | Months from lesion | Type of lesion | Fugl Meyer | Box of blocks R | Box of blocks L | Dynamometry R | Dynamometry L | Berg | Orpington |
| --- | --- | --- | --- | --- | --- | --- | --- | --- | --- | --- | --- | --- | --- |
| 1 | Co | 35 | M | R | 9 | I | 60.17 | 0 | 28 | 0 | 60 | 36 | 4.8 |
| 2 | Co | 51 | M | L | 18 | H | 79.64 | 27 | 12 | 12 | 48.3 | 38 | 3.2 |
| 3 | Co | 43 | F | L | 37 | H | 61.06 | 39 | 0 | 38.33 | 0 | 48 | 3.6 |
| 4 | Co | 47 | F | R | 19 | H | 87.16 | 13 | 46 | 0 | 48.3 | 55 | 3.2 |
| 5 | Co | 63 | F | L | 20 | H | 79.2 | 51 | 44 | 43.3 | 25 | 37 | 3.6 |
| 6 | Co | 58 | M | R | 7 | H | 47.34 | 0 | 36 | 0 | 21.6 | 18 | 6 |
| 7 | Co | 76 | M | L | 35 | I | 67.69 | 46 | 18 | 60 | 5 | 45 | 3.2 |
| 8 | Co | 70 | M | R | 204 | I | 82.74 | 19 | 21 | 20 | 10 | 42 | 2.8 |
| 9 | Co | 63 | M | L | 326 | I | 87.16 | 55 | 20 | 68.3 | 20.6 | 55 | 0 |
| 10 | Co | 53 | M | L | 2 | I | 90.26 | 18 | 17 | 50 | 23.3 | 25 | 3.2 |
| 11 | Ra | 55 | F | L | 10 | I | 67.2 | 29 | 14 | 23.3 | 0 | 41 | 4 |
| 12 | Ra | 35 | M | R | 25 | H | 44.24 | 0 | 18 | 0 | 52.3 | 32 | 4.8 |
| 13 | Ra | 52 | M | L | 24 | I | 98.67 | 42 | 37 | 83.33 | 57.33 | 49 | 2 |
| 14 | Ra | 44 | F | L | 6 | I | 66.81 | 62 | 0 | 46.66 | 0 | 43 | 4 |
| 15 | Ra | 61 | F | R | 27 | I | 65.92 | 0 | 52 | 15 | 50 | 56 | 4.4 |
| 16 | Ra | 38 | F | L | 2 | I | 71.23 | 37 | 0 | 38.3 | 0 | 48 | 1.2 |
| 17 | Ra | 59 | M | R | 16 | I | 80.53 | 0 | 44 | 18.3 | 63.3 | 48 | 2.8 |
| 18 | Ra | 56 | M | R | 5 | I | 77.8 | 28 | 64 | 41.6 | 65 | 56 | 2.8 |
| 19 | Ra | 59 | M | L | 252 | I | 54.4 | 47 | 0 | 50 | 0 | 36 | 4 |
| 20 | Ra | 73 | M | L | 8 | I | 69.4 | 42 | 13 | 23.3 | 16.6 | 50 | 3.2 |
| 21 | Ra | 48 | M | L | 44 | H | 63.2 | 42 | 13 | 60 | 0 | 48 | 2.8 |

Co: constant practice, Ra: random practice, Male, F: Female, R: Right, L: left, I: Ischemic, H: Hemorrhagic.
